# Sodium-Glucose Cotransporter-2 Inhibitors in Heart Failure with Malnutrition, Frailty, Sarcopenia, or Cachexia

**DOI:** 10.3390/jcm13061670

**Published:** 2024-03-14

**Authors:** Yu Horiuchi, Masahiko Asami, Kazuyuki Yahagi, Asahi Oshima, Yuki Gonda, Daiki Yoshiura, Kota Komiyama, Hitomi Yuzawa, Jun Tanaka, Jiro Aoki, Kengo Tanabe

**Affiliations:** Division of Cardiology, Mitsui Memorial Hospital, Tokyo 101-8643, Japanyuki.g17@gmail.com (Y.G.);

**Keywords:** heart failure, sodium-glucose cotransporter-2 inhibitors, malnutrition, frailty, sarcopenia, cachexia

## Abstract

(1) **Background**: In patients with heart failure (HF) and impaired nutritional status or decreased muscle mass, sodium-glucose cotransporter-2 inhibitors (SGLT2is) may worsen these conditions and result in poor prognosis, especially worsening of frailty. We aimed to investigate the relationship between SGLT2is and clinical outcomes, including frailty-related events, in patients with HF and malnutrition, frailty, sarcopenia, or cachexia. (2) **Methods**: In this retrospective observational cohort study, a global federated health research network provided data on patients with HF and malnutrition, frailty, sarcopenia, or cachexia from January 2016 to December 2021. We investigated the incidence of the composite endpoint of death or frailty-related events within one year. (3) **Results**: Among 214,778 patients included in the analysis, 4715 were treated with SGLT2is. After propensity score matching, 4697 patients in the SGLT2is group were matched with 4697 patients in the non-SGLT2is groups. The incidence of the composite endpoint, mortality, and frailty-related events was lower in the SGLT2is group than in the non-SGLT2is group (composite endpoint, 65.6% versus 77.6%, *p* < 0.001; mortality, 17.4% vs. 35.5%, *p* < 0.001; frailty-related events, 59.4% vs. 64.3%, *p* < 0.001). (4) **Conclusions**: Patients with HF and malnutrition, frailty, sarcopenia, or cachexia had a high incidence of death and frailty-related events. SGLT2is were associated with a lower incidence of these events.

## 1. Introduction

Frailty is a state of diminished homeostatic reserves across multiple physiological systems and increased susceptibility to internal and external stressors [1]. This condition is frequently, although not invariably, associated with aging and comorbidities, resulting in various detrimental health outcomes. Sarcopenia and cachexia often coexist with frailty and share the common feature of reduced muscle mass [2]. Heart failure (HF) is frequently complicated by frailty, sarcopenia, and cachexia, leading to a markedly elevated risk of functional decline, hospitalization, and mortality [3,4,5]. In patients with HF complicated by these conditions, the administration of new therapies is sometimes withheld. Due to concerns about a higher likelihood of adverse effects, reduced tolerance, poor adherence, and treatment discontinuation, new therapies may be seen as less efficacious [1,6].

Sodium-glucose cotransporter-2 inhibitors (SGLT2is) are glucose-lowering agents that decrease hyperglycemia by inhibiting SGLT2 in the proximal tubule of the kidney, which is responsible for the reabsorption of filtered glucose [7,8]. In patients with HF, SGLT2is have been shown to decrease the incidence of the composite endpoint of cardiovascular death or HF readmission, regardless of the presence or absence of diabetes, in both reduced and preserved left ventricular ejection fraction (LVEF) [9,10,11,12,13,14]. According to these results, the guidelines recommend the administration of SGLT2is for HF with reduced EF as a class I indication and for HF with preserved EF as a class I or IIa indication [15,16]. However, calorie loss caused by glucosuria may lead to the loss of lean body mass, especially fat and muscle loss [8]. Therefore, in patients with impaired nutritional reserve or decreased muscle mass, SGLT2is may worsen these conditions and result in poor prognosis, especially worsening of frailty. However, there are limited data regarding the administration of SGLT2is in patients with HF complicated by malnutrition, frailty, sarcopenia, or cachexia and their association with future frailty-related events.

This study aimed to investigate the relationship between SGLT2is and clinical outcomes, especially frailty-related events, in patients with HF and either malnutrition, frailty, sarcopenia, or cachexia, using a global healthcare research network.

## 2. Materials and Methods

### 2.1. Study Population

This retrospective observational study used a de-identified patient dataset from TrinetX (TriNetX, LLC., Cambridge, MA, USA), a global health-research database sourced and continuously updated from EHR. The TriNetX analytics platform has anonymous data from over 250 million patients across over 120 healthcare organizations in North and South America, Europe, the Middle East, Africa, and Asia–Pacific, including Japan. Most of these institutions are large and academic, covering a broad demographic spectrum. Users can access this platform online to execute queries, select cohorts, and compare outcomes. Data include longitudinal outpatient and in-hospital data and encompass patient demographics, medical diagnoses, laboratory test results, outpatient visits, hospitalizations, and mortality. Only aggregated data are reported without any identifiable personal health details. Even though the data are anonymized, TriNetX’s tools allow for patient-level statistical analysis [17,18,19]. A request can be submitted to TriNetX to access the data from the TriNetX research network. Access may incur costs and necessitate a data-sharing agreement.

This analysis included patients aged ≥ 18 years diagnosed with HF and malnutrition, frailty, sarcopenia, or cachexia from January 2016 to December 2021. Patients with estimated glomerular filtration rate (eGFR) < 15 mL/min/1.73 m^2^ or those undergoing hemodialysis were excluded because of the infrequent administration of SGLT2is [10,11,12,13]. ICD-10-CM codes represent diagnosis in TriNetX. If an organization provides data in ICD-9-CM, TriNetX utilizes a 9-to-10-CM mapping process based on general equivalence mappings, custom algorithms, and curation. The codes used for diagnoses are listed in Supplemental Table S1. The TriNetX network was searched on 20 February 2024, and an anonymized dataset from January 2016 was analyzed. At the time of the search, 113 participating healthcare organizations had data available for patients who met the study’s inclusion criteria. The SGLT2is group included patients treated with SGLT2is after diagnosis of HF and malnutrition, frailty, sarcopenia, or cachexia. Patients treated with SGLT2is before the diagnosis of these diseases were excluded. Considering the results of clinical trials and guideline recommendations, the SGLT2is group included patients treated with empagliflozin or dapagliflozin [15,16]. Patients treated with other SGLT2is were excluded. The non-SGLT2is group included patients with these diagnoses who were not treated with any type of SGLT2is. As a federated network, research studies utilizing the TriNetX network do not require ethical approval or patient informed consent, as they do not receive any patient-identifiable information.

### 2.2. Outcomes

The primary outcome was a composite of death or frailty-related events within one year. Frailty-related events were defined based on previous studies investigating ICD-10 codes related to frailty [20,21]. These codes are composed of seven domains: (1) dementia and delirium, (2) mobility problems, (3) falls and fractures, (4) pressure ulcer and weight loss, (5) incontinence, (6) dependence and care, and (7) anxiety and depression. The diagnoses and ICD-10 codes for these outcomes are shown in Supplemental Table S2. Time-to-event analysis began at the initiation of SGLT2is in the SGLT2is group and at the diagnosis of HF, malnutrition, frailty, sarcopenia, or cachexia in the non-SGLT2is group.

### 2.3. Statistical Analysis

All statistical analyses were performed using the TriNetX online platform. Baseline characteristics were compared using chi-square tests for categorical variables and independent sample *t*-tests for continuous variables. Propensity score (PS) matching was used to control for differences between the SGLT2is and non-SGLT2is groups. Patients in the SGLT2is and non-SGLT2is groups were 1:1 PS matched using logistic regression for age, sex, race, hypertensive diseases, ischemic heart disease, cerebral infarction, chronic kidney disease, diabetes mellitus, dyslipidemia, atrial fibrillation or flutter, anemia, overweight, obesity, malnutrition, frailty, sarcopenia, cachexia, systolic blood pressure, heart rate, body mass index, LVEF, hemoglobin A1c, eGFR, hemoglobin, B-type natriuretic peptide (BNP), N-terminal pro-B-type natriuretic peptide (NT-proBNP), and medications, including angiotensin-converting enzyme (ACE) inhibitors, angiotensin receptor blockers (ARBs), beta blockers, mineralocorticoid receptor antagonists (MRAs), loop diuretics, and antilipemic agents. These variables were chosen because they are known risk factors for HF or because there was a significant difference between the two groups [22,23,24,25,26]. The TriNetX platform employs input matrices of user-identified covariates and conducts logistic regression analysis to derive individual subjects’ PSs. One-to-one matching was executed using these scores, employing greedy nearest neighbor algorithms with a caliper width of 0.1 pooled standard deviations. Cohorts were considered well matched when the standardized mean difference was less than 0.1. Following PS matching, the Kaplan–Meier and Cox regression analyses were performed for outcomes. Subgroup analyses were conducted in HF patients with malnutrition and with frailty, sarcopenia, or cachexia. Subgroup analysis by types of SGLT2is and subgroups of patients with HF with reduced (LVEF < 40%), mildly reduced (40% ≤ LVEF < 50%) and preserved EF (LVEF ≥ 50%) were also investigated.

## 3. Results

A total of 214,778 patients with the diagnosis of HF and malnutrition, frailty, cachexia, or sarcopenia were included in the analysis: the SGLT2is group included 4715 patients, and the non-SGLT2is group included 210,063 patients.

Table 1 shows the baseline characteristics of the SGLT2is and non-SGLT2is groups. Before PS matching, patients in the SGLT2is group were younger, more likely to be men and African American, and less likely to be Caucasians than those in the non-SGLT2is group. The SGLT2is group was more frequently associated with comorbidities, including hypertensive disease, ischemic heart disease, cerebral infarction, chronic kidney disease, diabetes mellitus, dyslipidemia, atrial fibrillation or flutter, and anemia. Overweight, malnutrition and sarcopenia were more common, and frailty and cachexia were less commonly observed in the SGLT2is group. Body mass index (BMI) was higher in the SGLT2is group. Patients in the SGLT2is group had lower LVEF and NT-proBNP values and higher hemoglobin A1c and BNP values than patients in the non-SGLT2is group. Medications for HF, such as ACE inhibitors, ARBs, beta blockers, MRAs, and loop diuretics, were more frequently used in the SGLT2is group than in the non-SGLT2is group. After PS matching, 4697 patients in the SGLT2is group were matched with 4697 patients in the non-SGLT2is groups (Table 2). Baseline characteristics were comparable with standardized mean difference < 0.1, except for hemoglobin A1c and eGFR values. PS density showed concordance in both groups (Supplemental Figure S1).

**Figure 1:**
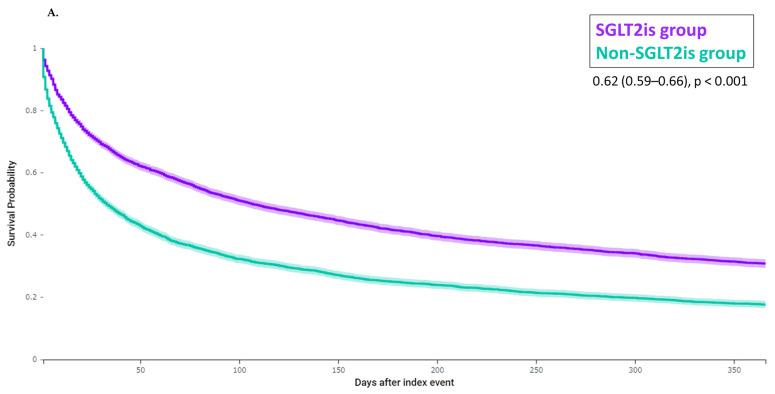

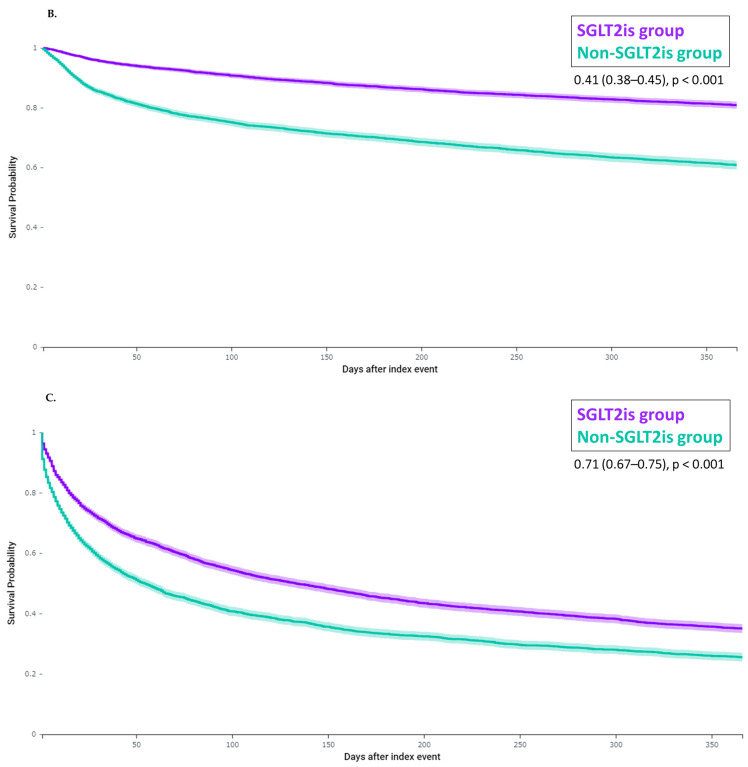
(**A**) Kaplan–Meier analysis for the composite outcome of death or frailty-related events; (**B**) Kaplan–Meier analysis for death; (**C**) Kaplan–Meier analysis for frailty-related events. After PSM, the SGLT2is group (purple line) compared to the non-SGLT2is group (green line) was associated with lower HRs for the composite outcome of death or frailty-related events, death and frailty-related events within one year. HR: hazard ratio; PSM: propensity score matching; SGLT2is: sodium-glucose transporter 2 inhibitors.

**Figure 2:**
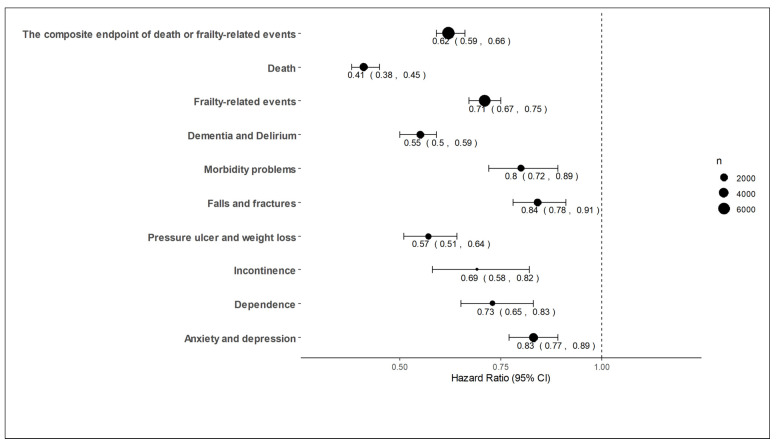
Hazard ratios for components of frailty-related events. HRs of the SGLT2is group referenced to the non-SGLT2is group for the components of frailty-related events within one year are shown in the figure. The SGLT2is group was associated with lower HRs for each component of frailty-related events. HR: hazard ratio; SGLT2is: sodium-glucose transporter 2 inhibitors.

**Figure 3:**
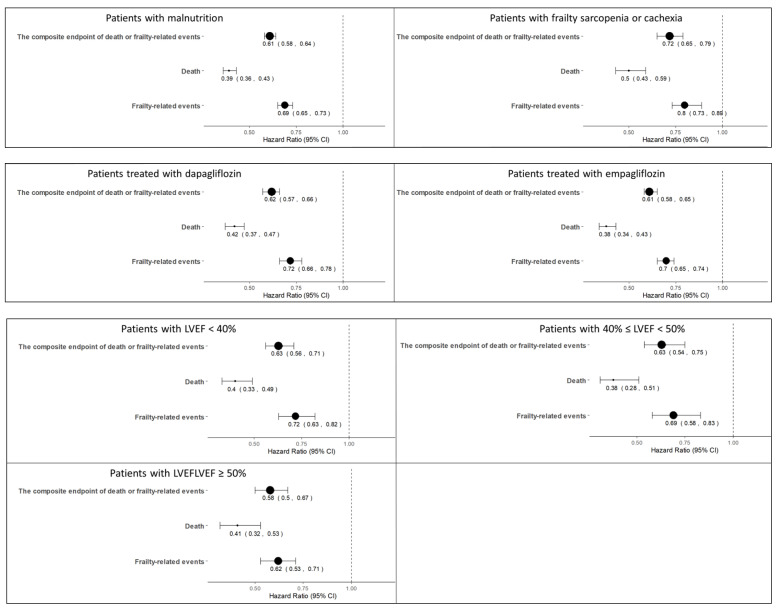
Hazard ratios for the composite endpoint, death and frailty-related events. HRs of the SGLT2is group referenced to the non-SGLT2is group are shown in the figure. After PSM, the SGLT2is group was associated with lower HRs for the composite endpoint, death, and frailty-related events in patients with malnutrition and those with frailty, sarcopenia or cachexia. Findings were similar in dapagliflozin and empagliflozin groups, and in HFrEF, HFmrEF and HFpEF.

The composite endpoint, death and frailty-related events were observed in 6723, 2485, and 5812 patients, respectively. The incidence of the composite endpoint was lower in the SGLT2is group than in the non-SGLT2is group (65.6% versus 77.6%, hazard ratio [HR] 0.62 [0.59–0.66], *p* < 0.001, Figure 1A). Mortality and the incidence of frailty-related events were also lower in the SGLT2is group than in the non-SGLT2is group (mortality, 17.4% vs. 35.5%, HR 0.41 [0.38–0.45], *p* < 0.001; frailty-related events, 59.4% vs. 64.3%, HR 0.71 [0.67–0.75], *p* < 0.001, Figure 1B,C). Regarding each component of frailty-related events, dementia and delirium were observed in 2203, morbidity problems in 1519, falls and fractures in 2299, pressure ulcer and weight loss in 1324, incontinence in 480, dependence in 952, and anxiety and depression in 3374 patients. The SGLT2is group was associated with lower HRs for each component of frailty-related events (Figure 2). The number of events and incidence of all diagnoses of frailty-related events are shown in Supplemental Table S3. Similar results were observed in subgroup analyses (Figure 3). After PS matching, the SGLT2is group was associated with lower HRs of the composite endpoint, death, and frailty-related events in patients with malnutrition (4007 in SGLT2is and non-SGLT2 groups, respectively) and patients with frailty, sarcopenia or cachexia (1255 in SGLT2is and non-SGLT2 groups, respectively). When SGLT2is were analyzed by type, both dapagliflozin (2032 patients) and empagliflozin (2889 patients) were associated with a lower incidence of the composite endpoint. Lower HRs in the SGLT2is group were also observed in patients with HFrEF (691 patients in SGLT2is and non-SGLT2 groups, respectively), HFmrEF (383 patients in each group) and HFpEF (534 patients in each group).

## 4. Discussion

This study investigated the relationship between SGLT2is and the composite endpoint of death or frailty-related events in patients with HF complicated by malnutrition, frailty, sarcopenia, or cachexia. Patients treated with SGLT2is were more frequently associated with comorbidities and more often treated with medical therapies for HF than those not treated with SGLT2is. After PS matching, the incidence of the composite endpoint, death, and frailty-related events was lower in the SGLT2is group than in the non-SGLT2is group. These results were consistent in the subgroups of patients with malnutrition and those with frailty, sarcopenia, or cachexia.

In patients with HF, the efficacy of SGLT2is on mortality and HF hospitalization is reported to be consistent, regardless of age or BMI [27,28,29]. However, these findings do not necessarily support its benefit in frail patients because frailty cannot be assessed only by age or BMI. Subanalyses of the DAPA-HF and DELIVER trials evaluated the efficacy of dapagliflozin in frail patients using the Rockwood cumulative deficit analysis of the 32-item frailty index [30,31]. Almost 50% of the patients with LVEF ≤ 40% and 62.4% of the patients with LVEF > 40% were diagnosed with frailty. The effect of dapagliflozin on the composite endpoint of cardiovascular death or HF hospitalization was not affected by frailty severity. Our analysis adds to the literature that SGLT2is may be beneficial in malnourished HF patients who are at risk of frailty due to SGLT2is-induced calorie loss, decreased fat and muscle waste, as well as those who are already complicated by frailty, sarcopenia, or cachexia.

Another novelty of this study was the association between SGLT2is and frailty-related events. Based on previous studies, seven groups of outcomes were chosen to represent the common domains of frailty [20,21]. These events were commonly observed in the study population. In the non-SGLT2is group after the PS matching, 64.3% experienced frailty-related events within one year. Patients in the SGLT2is group had a lower incidence of frailty-related events and all its domains. SGLT2is reduces the ratio of insulin to glucagon and enhances hepatic gluconeogenesis, which may lead to the loss of fat and muscle mass [32]. Several studies have reported decreased skeletal muscle mass and weakness with SGLT2is in patients with type 2 diabetes mellitus [33,34]. However, another study reported that SGLT2is improved muscle strength, gait speed, and 6 min walk distance [35]. In patients with HF, SGLT2is have been shown to improve functional capacity, as assessed by peak VO2 [36,37]. Improvements in cardiopulmonary functional capacity may result in a lower incidence of frailty-related events, as shown in the results of our analysis. Importantly, even in the SGLT2is group, frailty-related events were as high as 59.4% within one year. It should be noted that HF patients with malnutrition, frailty, sarcopenia, or cachexia are at risk of frailty-related events, even after treatment with SGLT2is.

Cognitive dysfunction and an impaired mental status are essential components of frailty. In this study, SGLT2is were associated with an improvement of these frailty domains. This may not be a direct effect of SGLT2i and can be an indirect impact on improved subjective symptoms. Improvement in quality of life, as assessed by the Kansas City Cardiomyopathy Questionnaire, has been observed very early after the initiation of SGLT2is [38,39,40]. Considering the close relationship between physical and cognitive frailty, improved physical function may also contribute to better cognitive function and mental status [41].

The strength of this study lies in its use of extensive international clinical data. In the context of limited data on malnutrition and frailty among patients with HF, this study specifically targeted patients with these comorbidities undergoing treatment with SGLT2is. Furthermore, distinctive endpoints were evaluated to assess the progression of frailty.

### Limitations

The electronic healthcare record was used in the study, and all diagnoses were based on the ICD-10 codes. The diagnosis of malnutrition, frailty, sarcopenia, and cachexia is not defined. Some values were missing, especially in the laboratory measurements. About three-quarters of the patients lacked LVEF measurement, although the efficacy of SGLT2is is not affected by LVEF [14]. While we included as many important prognostic factors as possible in PS matching, unknown and unmeasured confounders cannot be adjusted. Therefore, the results are only hypothesis-generating. A comprehensive assessment of physical frailty, such as a short physical performance battery or the Fried phenotype model, was unavailable. Direct muscle strength or mass measurements, such as grip strength and dual-energy X-ray absorptiometry, were not recorded. The values of BNP and NT-proBNP were missing in many patients, while at least one of these biomarkers was assessed in the majority of patients after PS matching.

Future studies should utilize standardized definitions for malnutrition, frailty, sarcopenia, and cachexia to investigate the impact of SGLT2is on the progression of frailty in these patients. By incorporating comprehensive assessments of physical frailty and direct measurements of muscle strength or mass, our understanding of malnutrition and frailty in patients with HF and their treatment with SGLT2is will be significantly enhanced.

## 5. Conclusions

Patients with HF and malnutrition, frailty, sarcopenia, or cachexia were associated with a high incidence of death and frailty-related events. The administration of SGLT2is was associated with a lower incidence of these events. SGLT2is may be beneficial in patients with HF and impaired nutritional status or decreased muscle mass.

## Figures and Tables

**Table 1:** Baseline characteristics before propensity score matching.

	SGLT2is Group (n = 4715)	% of Missing Values	Non-SGLT2is Group (n = 210,063)	% of Missing Values	Standardized Mean Difference
Demographics					
Age (years)	67 ± 12	0%	73 ± 14	0%	0.496
Male	2805 (60%)	0%	99,027 (47%)	0%	0.249
Race		0%		0%	
Caucasians	2734 (58%)		140,463 (67%)		0.184
Black or African American	944 (20%)		29,350 (14%)		0.162
Comorbidities					
Hypertensive diseases	4500 (95%)	0%	185,863 (89%)	0%	0.258
Ischemic heart diseases	4005 (85%)	0%	136,591 (65%)	0%	0.473
Cerebral infarction	985 (21%)	0%	34,655 (17%)	0%	0.113
Chronic kidney disease	2849 (60%)	0%	94,427 (45%)	0%	0.314
Diabetes mellitus	3862 (82%)	0%	87,811 (42%)	0%	0.907
Dyslipidaemia	4049 (86%)	0%	140,823 (67%)	0%	0.455
Atrial fibrillation and flutter	2538 (54%)	0%	104,355 (50%)	0%	0.083
Anemia	2958 (63%)	0%	113,066 (54%)	0%	0.181
Overweight and obesity	2342 (50%)	0%	55,675 (27%)	0%	0.491
Malnutrition	3964 (84%)	0%	167,115 (80%)	0%	0.117
Frailty	683 (15%)	0%	32,879 (16%)	0%	0.033
Sarcopenia	64 (1.4%)	0%	1634 (0.80%)	0%	0.056
Cachexia	453 (9.6%)	0%	28,590 (14%)	0%	0.125
ICD	1162 (25%)	0%	14,294 (6.8%)	0%	0.505
CRTD	179 (3.8%)	0%	1296 (0.6%)	0%	0.218
Physical findings					
Systolic Blood Pressure (mmHg)	117 ± 23	27%	119 ± 26	34%	0.059
Heart rate (bpm)	78 ± 16	27%	78 ± 18	35%	0.012
BMI (kg/m^2^)	28.7 ± 7.4	53%	25.8 ± 6.9	62%	0.403
LVEF (%)	39.7 ± 18.0	77%	52.6 ± 16.3	84%	0.75
Laboratory values					
Haemoglobin A1c (%)	7.4 ± 2.0	13%	6.3 ± 1.7	55%	0.592
eGFR (mL/min/1.73 m^2^)	65 ± 30	0%	64 ± 35	0%	0.041
BNP (pg/mL)	1420 ± 3924	48%	1234 ± 4316	47%	0.045
NT-proBNP (pg/mL)	4492 ± 7555	55%	5553 ± 9011	71%	0.128
Medications					
ACE-inhibitors	3132 (66%)	0%	84,971 (41%)	0%	0.539
ARBs	3021 (64%)	0%	52,301 (25%)	0%	0.858
Beta blockers	4451 (94%)	0%	153,728 (73%)	0%	0.601
MRAs	2759 (59%)	0%	35,894 (17%)	0%	0.945
Loop diuretics	4320 (92%)	0%	144,291 (69%)	0%	0.6
Antilipemic agents	4232 (90%)	0%	130,262 (62%)	0%	0.686

ACE: angiotensin-converting enzyme; ARB: angiotensin receptor blockers; BMI: body mass index; BNP: B-type natriuretic peptide; CRTD: cardiac resynchronization therapy defibrillator; eGFR: estimated glomerular filtration rate; ICD: implantable cardiac defibrillator; LVEF: left ventricular ejection fraction; NT-proBNP: N-terminal pro B-type natriuretic peptide; MRAs: mineralocorticoid receptor antagonists; SGLT2is: sodium-glucose transporter 2 inhibitors.

**Table 2:** Baseline characteristics after propensity score matching.

	SGLT2is Group (n = 4697)	% of Missing Values	Non-SGLT2is Group (n = 4697)	% of Missing Values	Standardized Mean Difference
Demographics					
Age (years)	67 ± 12	0%	67 ± 14	0%	0.028
Male	2792 (59%)	0%	2787 (59%)	0%	0.002
Race		0%		0%	
Caucasians	2726 (58%)		2737 (58%)		0.005
Black or African American	939 (20%)		922 (20%)		0.009
Comorbidities					
Hypertensive diseases	4482 (950%)	0%	4493 (96%)	0%	0.011
Ischemic heart diseases	3988 (85%)	0%	3986 (85%)	0%	0.001
Cerebral infarction	980 (21%)	0%	948 (20%)	0%	0.017
Chronic kidney disease	2833 (60%)	0%	2804 (60%)	0%	0.013
Diabetes mellitus	3844 (82%)	0%	3929 (84%)	0%	0.048
Dyslipidaemia	4031 (86%)	0%	4071 (87%)	0%	0.025
Atrial fibrillation and flutter	2527 (54%)	0%	2541 (54%)	0%	0.006
Anemia	2948 (63%)	0%	2954 (63%)	0%	0.003
Overweight and obesity	2329 (50%)	0%	2368 (50%)	0%	0.017
Malnutrition	3947 (84%)	0%	3962 (84%)	0%	0.009
Frailty	680 (15%)	0%	678 (14%)	0%	0.001
Sarcopenia	64 (1.40%)	0%	77 (1.6%)	0%	0.023
Cachexia	453 (9.6%)	0%	419 (8.9%)	0%	0.025
ICD	1146 (24%)	0%	1109 (24%)	0%	0.018
CRTD	176 (3.7%)	0%	167 (3.6%)	0%	0.01
Physical findings					
Systolic Blood Pressure (mmHg)	118 ± 23	27%	115 ± 26	28%	0.088
Heart rate (bpm)	78 ± 16	27%	79 ± 18	25%	0.042
BMI (kg/m^2^)	28.7 ± 7.4	53%	28.3 ± 7.3	52%	0.058
LVEF (%)	39.8 ± 18.1	77%	40.7 ± 18.6	76%	0.049
Laboratory values					
Haemoglobin A1c (%)	7.4 ± 2.0	13%	7.2 ± 2.1	14%	0.126
eGFR (mL/min/1.73 m^2^)	64.8 ± 30.4	0%	61.5 ± 33.2	0%	0.104
BNP (pg/mL)	1422 ± 3929	48%	1421 ± 4236	47%	<0.001
NT-proBNP (pg/mL)	4482 ± 7557	55%	4998 ± 8200	56%	0.065
Medications					
ACE-inhibitors	3115 (66%)	0%	3144 (67%)	0%	0.013
ARBs	4433 (94%)	0%	4463 (95%)	0%	0.012
Beta blockers	4433 (94%)	0%	4463 (95%)	0%	0.029
MRAs	2741 (58%)	0%	2694 (57%)	0%	0.02
Loop diuretics	4302 (92%)	0%	4312 (92%)	0%	0.008
Antilipemic agents	4214 (90%)	0%	4255 (91%)	0%	0.029

ACE: angiotensin-converting enzyme; ARB: angiotensin receptor blockers; BMI: body mass index; BNP: B-type natriuretic peptide; CRTD: cardiac resynchronization therapy defibrillator; eGFR: estimated glomerular filtration rate; ICD: implantable cardiac defibrillator; LVEF: left ventricular ejection fraction; NT-proBNP: N-terminal pro B-type natriuretic peptide; MRAs: mineralocorticoid receptor antagonists; SGLT2is: sodium-glucose transporter 2 inhibitors.

## Data Availability

A request can be submitted to TriNetX to access the data from the TriNetX research network.

## Supplementary Materials

The following supporting information can be downloaded at: https://www.mdpi.com/article/10.3390/jcm13061670/s1, Figure S1: Propensity score density; Table S1: ICD-10 codes used in the analysis; Table S2: Diagnosis in each domain of frailty-related events; Table S3: Incidence of frailty-related events.
