# Supplementary material for: Sodium-Glucose Cotransporter-2 Inhibitors in Heart Failure with Malnutrition, Frailty, Sarcopenia, or Cachexia
Source: J Clin Med. 2024 Mar 14;13(6):1670. doi: 10.3390/jcm13061670 (PMC10970728; doi:10.3390/jcm13061670)
Supplement: Supplementary file 1 [file jcm-13-01670-s001.zip › Supplementary_Figure_R1.pdf]

Supplemental Figure. Propensity-score density before and after the matching

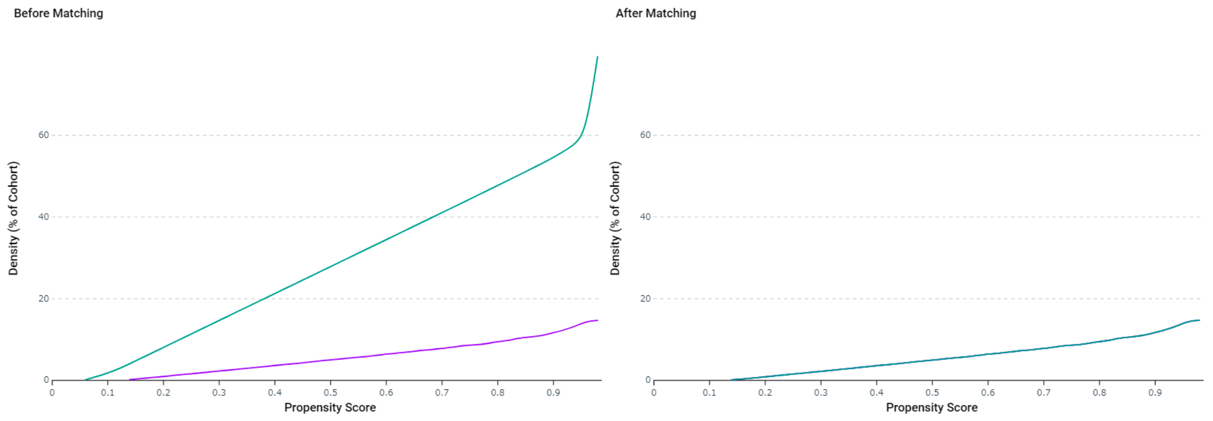

After PSM, 4,697 patients in the SGLT2is group were matched with 4,697 patients in the non-SGLT2is group. Propensity score density showed concordance in the SGLT2is (purple line) and non-SGLT2is (green line) groups.

PSM, propensity score matching; SGLT2is, sodium-glucose transporter 2 inhibitors
