# Supplementary material for: Sodium-Glucose Cotransporter-2 Inhibitors in Heart Failure with Malnutrition, Frailty, Sarcopenia, or Cachexia
Source: J Clin Med. 2024 Mar 14;13(6):1670. doi: 10.3390/jcm13061670 (PMC10970728; doi:10.3390/jcm13061670)
Supplement: Supplementary file 1 [file jcm-13-01670-s001.zip › Supplementary_Table_R1.pdf]

Supplemental Table 1. ICD-10 codes used in the analysis	
D64	Anemia
E08-E13	Diabetes mellitus
E40-E46	Malnutrition
E66	Overweight and obesity
E78	Disorders of lipoprotein metabolism and other lipidemias
F01	Vascular dementia
F02	Dementia in other diseases classified elsewhere
F03	Unspecified dementia
F04	Amnestic disorder due to known physiological condition
F05	Delirium due to known physiological condition
F06.4	Anxiety disorder due to known physiological condition
F25.1	Schizoaffective disorder, depressive type
F31	Bipolar disorder
F32	Depressive episode
F33	Major depressive disorder, recurrent
F34.1	Dysthymic disorder
F41	Other anxiety disorders
F43	Reaction to severe stress, and adjustment disorders
F43.2	Adjustment disorders
F44	Dissociative and conversion disorders
G30	Alzheimer's disease
G31.0	Frontotemporal dementia
G31.1	Senile degeneration of brain, not elsewhere classified
I10-I1A	Hypertensive diseases
I20-I25	Ischemic heart diseases
I48	Atrial fibrillation and flutter
I50	Heart failure
I63	Cerebral infarction
L89	Pressure ulcer
M62.84	Sarcopenia
M80	Osteoporosis with current pathological fracture
M81	Osteoporosis without current pathological fracture
M96.6	Fracture of bone following insertion of orthopedic implant, joint prosthesis, or bone plate
N18	Chronic kidney disease
R15	Fecal incontinence
R26	Abnormalities of gait and mobility
R29.6	Repeated falls
R29.8	Other symptoms and signs involving the nervous and musculoskeletal systems

R32	Unspecified urinary incontinence
R41	Other symptoms and signs involving cognitive functions and awareness
R54	Age-related physical debility (frailty)
R55	Syncope and collapse
R63.4	Abnormal weight loss
R63.6	Underweight
R64	Cachexia
S32	Fracture of lumbar spine and pelvis
S33	Dislocation and sprain of joints and ligaments of lumbar spine and pelvis
S42	Fracture of shoulder and upper arm
S43	Dislocation and sprain of joints and ligaments of shoulder girdle
S52	Fracture of forearm
S53	Dislocation and sprain of joints and ligaments of elbow
S62	Fracture at wrist and hand level
S63	Dislocation and sprain of joints and ligaments at wrist and hand level
S72	Fracture of femur
S73	Dislocation and sprain of joint and ligaments of hip
W00-W19	Slipping, tripping, stumbling and falls
Z72.4	Inappropriate diet and eating habits
Z74	Problems related to care provider dependency
Z75	Problems related to medical facilities and other health care

Supplemental Table 2. Diagnosis in each domain of frailty-related events	
**1. Dementia and Delirium**	Vascular dementia
	Dementia in other diseases classified elsewhere
	Unspecified dementia
	Delirium not induced by alcohol and other psychoactive
	Alzheimer's disease
	Senile degeneration of brain, not elsewhere classified
	Circumscribed brain atrophy
	Organic amnesic syndrome, not induced by alcohol and other psychoactive substances
	Other symptoms and signs involving cognitive functions and awareness
	Abnormalities of gait and mobility
**2. Mobility Problems**	Other and unspecified symptoms and signs involving the nervous and musculoskeletal systems
	Fracture of lumbar spine and pelvis
**3. Falls and Fractures**	Dislocation, sprain and strain of joints and ligaments of lumbar spine and pelvis
	Fracture of shoulder and upper arm
	Dislocation, sprain and strain of joints and ligaments of shoulder girdle
	Fracture of forearm
	Dislocation, sprain and strain of joints and ligaments of elbow
	Fracture at wrist and hand level
	Dislocation, sprain and strain of joints and ligaments at wrist and hand level
	Fracture of femur
	Dislocation, sprain and strain of joint and ligaments of hip
	Falls
	Osteoporosis with pathological fracture
	Osteoporosis without pathological fracture
	Tendency to fall, not elsewhere classified
	Syncope and collapse
	Fracture of bone following insertion of orthopedic implant, joint prosthesis, or bone plate
	Decubitus ulcer and pressure area
**4. Pressure Ulcers and Weight Loss**	Abnormal weight loss
	Insufficient intake of food and water due to self-neglect
	Inappropriate diet and eating habits
	Unspecified urinary incontinence

**5. Incontinence**	Fecal incontinence
	Problems related to care-provider dependency
**6. Dependence and Care**	Problems related to medical facilities and other healthcare
**7. Anxiety and Depression**	Other anxiety disorders
	Reaction to severe stress, and adjustment disorders
	Dissociative [conversion] disorders
	Organic anxiety disorder
	Depressive episode
	Recurrent depressive disorder
	Schizoaffective disorder, depressive type
	Bipolar affective disorder
	Dysthymia
	Adjustment disorders

Supplemental Table 3. Incidence of frailty-related events			
		SGLT2is group (n = 4,697)	Non-SGLT2is group (n = 4,697)
**1. Dementia and Delirium**	Vascular dementia	62 (1.3%)	95 (2%)
	Dementia in other diseases classified elsewhere	70 (1.5%)	116 (2.5%)
	Unspecified dementia	178 (3.8%)	273 (5.8%)
	Delirium not induced by alcohol and other psychoactive	125 (2.7%)	220 (4.7%)
	Alzheimer's disease	52 (1.1%)	67 (1.4%)
	Senile degeneration of brain, not elsewhere classified	10 (0.2%)	10 (0.2%)
	Circumscribed brain atrophy	10 (0.2%)	10 (0.2%)
	Organic amnesic syndrome, not induced by alcohol and other psychoactive substances	10 (0.2%)	10 (0.2%)
	Other symptoms and signs involving cognitive functions and awareness	735 (15.6%)	1101 (23.4%)
**2. Mobility Problems**	Abnormalities of gait and mobility	520 (11.1%)	514 (10.9%)
	Other and unspecified symptoms and signs involving the nervous and musculoskeletal systems	326 (6.9%)	380 (8.1%)
	Fracture of lumbar spine and pelvis	92 (2%)	117 (2.5%)
**3. Falls and Fractures**	Dislocation, sprain and strain of joints and ligaments of lumbar spine and pelvis	10 (0.2%)	10 (0.2%)
	Fracture of shoulder and upper arm	44 (0.9%)	62 (1.3%)
	Dislocation, sprain and strain of joints and ligaments of shoulder girdle	24 (0.5%)	15 (0.3%)
	Fracture of forearm	22 (0.5%)	21 (0.4%)

	Dislocation, sprain and strain of joints and ligaments of elbow	10 (0.2%)	0 (0%)
	Fracture at wrist and hand level	25 (0.5%)	25 (0.5%)
	Dislocation, sprain and strain of joints and ligaments at wrist and hand level	14 (0.3%)	10 (0.2%)
	Fracture of femur	85 (1.8%)	107 (2.3%)
	Dislocation, sprain and strain of joint and ligaments of hip	10 (0.2%)	10 (0.2%)
	Falls	414 (8.8%)	450 (9.6%)
	Osteoporosis with pathological fracture	53 (1.1%)	55 (1.2%)
	Osteoporosis without pathological fracture	332 (7.1%)	299 (6.4%)
	Tendency to fall, not elsewhere classified	218 (4.6%)	222 (4.7%)
	Syncope and collapse	394 (8.4%)	369 (7.9%)
	Fracture of bone following insertion of orthopedic implant, joint prosthesis, or bone plate	0 (0%)	10 (0.2%)
	Decubitus ulcer and pressure area	323 (6.9%)	582 (12.4%)
**4. Pressure Ulcers and Weight Loss**	Abnormal weight loss	220 (4.7%)	222 (4.7%)
	Insufficient intake of food and water due to self-neglect	11 (0.2%)	15 (0.3%)
	Inappropriate diet and eating habits	10 (0.2%)	10 (0.2%)
	Unspecified urinary incontinence	177 (3.8%)	218 (4.6%)
**5. Incontinence**	Faecal incontinence	70 (1.5%)	77 (1.6%)
	Problems related to care-provider dependency	427 (9.1%)	497 (10.6%)
**6. Dependence and Care**	Problems related to medical facilities and other healthcare	20 (0.4%)	28 (0.6%)
**7. Anxiety and Depression**	Other anxiety disorders	988 (21%)	1011 (21.5%)

	Reaction to severe stress, and adjustment disorders	273 (5.8%)	286 (6.1%)
	Dissociative [conversion] disorders	15 (0.3%)	20 (0.4%)
	Organic anxiety disorder	25 (0.5%)	28 (0.6%)
	Depressive episode	1075 (22.9%)	1129 (24%)
	Recurrent depressive disorder	298 (6.3%)	253 (5.4%)
	Schizoaffective disorder, depressive type	10 (0.2%)	10 (0.2%)
	Bipolar affective disorder	100 (2.1%)	101 (2.2%)
	Dysthymia	41 (0.9%)	46 (1%)
	Adjustment disorders	174 (3.7%)	199 (4.2%)
